# The Robustness of Anchoring in a Naturalistic VR‐Based Task

**DOI:** 10.1111/cogs.70229

**Published:** 2026-06-15

**Authors:** Jinjin Wu, George Farmer, Olivia Pready‐James, Paul A. Warren

**Affiliations:** ^1^ Virtual Reality Research (VR2) Facility University of Manchester; ^2^ Perception Action and Decision Laboratory (PADLab) University of Manchester; ^3^ Andrew Mayes Centre for Cognitive Neuroscience University of Manchester; ^4^ Division of Psychology Communication and Human Neuroscience University of Manchester

**Keywords:** Judgment, Decision making, Anchoring, Perception, Cognition, Virtual reality

## Abstract

Anchoring occurs when a quantitative estimate is biased toward an initially presented value (the anchor). Anchoring occurs both in high‐level explicit estimation of numeric quantities and in lower‐level perceptual tasks and persists even when reliable information about the quantity being estimated is directly available at the point of judgment. This suggests anchoring might derive from generic processing underpinning estimation. Such tasks, however, are almost exclusively lab‐based, and the information required to complete the task is rarely available to the participant in a way that reflects how such information is sampled in real‐life. To address this, across two experiments, we immersed participants in a virtual world on a platform that could be placed at any height. After an anchor was presented, participants subsequently estimated their height naturally, by interpreting sensory and cognitive cues from the 3D environment in which they were immersed. We manipulated the amount of information directly available to the participant for making their judgment as well as the offset between the anchor and the actual height. To modulate the extent to which task‐relevant information was acquired naturally from the environment, we also contrasted anchoring effects in and out of VR. Although participants clearly used the available perceptual and cognitive information to make height estimates, anchoring effects were evident, displayed similar properties to those reported in previous lab‐based studies, and were consistent both in and out of VR. Our design also allowed us to recover a novel subjective anchoring measure that facilitated a particularly parsimonious descriptive model of our anchoring data. We conclude that anchoring is a generic feature of all estimation tasks, even when task‐relevant information is acquired naturally, as in real‐life estimation. These results emphasize the potential for this cognitive bias to have a significant impact on performance in any real‐world task requiring quantitative estimation.

## 1 Introduction

Decades of research, dating back to the seminal work of Amos Tversky and Daniel Kahneman (1974), have focused on identifying and investigating behavioral phenomena in which humans exhibit systematic departures from rational judgment and decision making (JDM). There has been considerable work on the potential causes of such *cognitive bias* phenomena including a good deal of work on the extent to which such behaviors should even be characterized as biased (Hahn & Warren, 2009; Howes, Warren, Farmer, El‐Deredy, & Lewis, 2016; Oaksford & Chater, 1998). Nonetheless, such behavioral phenomena exist and offer the opportunity to gain insight on the cognitive processes of human JDM, in the same way that visual illusions have enabled researchers to learn a great deal about the sensory and perceptual processing that underpins human vision.

One simple argument that might make us question the idea of cognitive bias is that such phenomena are often encountered in experimental tasks that are abstract, hypothetical, inconsequential, and unnatural. If we ask people to take part in such experiments, which are typically quite different from the kinds of tasks we might have evolved to undertake, then it does not seem particularly surprising that seemingly irrational behaviors emerge (Allen et al., 2024; Simon, 1972). These behaviors may arise as a simple by‐product of optimizing behavior on some other metric the experimenter is unaware of. There is at least some evidence that when cognitive bias tasks are placed in a more natural context, biases are reduced or even disappear completely (Frederick, Lee, & Baskin, 2014; Trendl, Stewart, & Mullett, 2021). One way to potentially address this issue is to conduct experiments in the real‐world and use tasks that are more natural, well‐constrained and unambiguous to the participant. Unfortunately, real‐world experimentation is, of course, problematic due to unavoidable issues of experimental control. However, the recent advent of reliable and near lag‐free virtual reality (VR) offers an exciting opportunity to develop naturalistic JDM tasks in well‐controlled (and safe) environments and re‐examine previously identified cognitive bias phenomena.

In the present study, we use VR to study a particularly well‐examined and robust cognitive bias—anchoring. Anchoring describes a tendency in human JDM to be biased toward an initially provided (usually numeric) piece of information (Tversky & Kahneman, 1974). A common anchoring paradigm consists of an initial comparative question (CQ), relative to an anchor and a subsequent estimation question (EQ). For example, participants might first answer a CQ such as: “Is Mount Everest higher or lower than 45,500 feet?” before being asked to provide an estimate in response to an EQ such as: “How high is Mount Everest?” (Jacowitz & Kahneman, 1995). Typically, in a between‐subjects design, half of the participants are exposed to the high anchor (45,500 feet), and half are exposed to a low anchor (e.g., 2000 feet). Subsequent estimates are typically biased toward whichever anchor participants have been exposed to. It should be emphasized that classical cognitive anchoring paradigms usually implicate “offline” information—that is, the estimates to be made typically involve the use of stored knowledge (e.g., the height of Mount Everest) or making an estimate of a quantity that is difficult to estimate (number of doctors in the phone book) as opposed to making an estimate based on some currently available information from which the answer could be derived directly. Given that humans must regularly make estimates based on currently available information, it seems important to know if anchoring would persist in such circumstances. For example, if asked to judge the speed of an oncoming vehicle when crossing the road from visual and/or auditory cues.

This latter example is interesting as it represents a perceptual judgment. Recent research has found evidence for anchoring effects in lower‐level perceptual judgments (Jain, Nayakankuppam, & Gaeth, 2021; LeBoeuf & Shafir, 2006; Spicer et al., 2022). Anchoring has been reported for a range of perceptual modalities including estimates of numerosity (Spicer et al., 2022), weight, length, and loudness (LeBoeuf & Shafir, 2006) as well as coarseness, frequency, and luminance (Jain et al., 2021). These results are at least consistent with the idea that anchoring to an initial numeric quantity is a generic property of any estimation task, irrespective of whether this involves lower‐level perceptual or higher‐level cognitive processing. It is important to note that the tasks used in these perceptual anchoring studies are similar to those in higher‐level anchoring studies, in the sense that they often rely upon storing knowledge about a quantity before making a subsequent estimate, that is, they depend upon offline estimation and comparison with a stored or memorized stimulus. In other words, they are rather different in nature from the example given above of estimating the speed of an approaching car using directly available sensory information. One might argue therefore that these effects are driven in part by not having online access to the information required to complete the task. However, there is also research to suggest that anchoring even persists in such circumstances (e.g., Garcia‐Marques & Fernandes, 2023; Langeborg & Eriksson, 2016; Lee & Morewedge, 2022; Sawyer & Wesensten, 1994). For example, Lee and Morewedge (2022) asked two groups of participants to estimate the number of dots in a scatter plot after first making a comparative judgment about whether the quantity was more or less than a low (Group 1) or high (Group 2) anchor. Estimates were systematically shifted toward the anchor even though the estimate to be derived was based on information that was directly and immediately available to the participant. This suggests that anchoring is a particularly robust cognitive bias phenomenon—it persists even when information required to make an accurate estimate is available to the participant.

Several factors that act to modulate anchoring effects have also been investigated. For example, the magnitude of the anchor offset (from an objective correct answer) has been shown to affect the size of the anchoring effect (e.g., see Mussweiler & Strack, 2001). More specifically, the effect increases with offset but then decreases as the offset gets too large, presumably because the anchor becomes less plausible (Mussweiler & Strack, 2001; Teovanović, 2019; Wegener, Petty, Detweiler‐Bedell, & Jarvis, 2001). There is also evidence for asymmetries in anchoring such that positive anchor offsets result in larger anchoring than negative anchor offsets of the same absolute value (Hardt & Pohl, 2003; Jasper & Christman, 2005; Teovanović, 2019). In addition, knowledge/familiarity with the EQ was found to attenuate the anchoring effect (Mussweiler & Strack, 2000; Smith, Windschitl, & Bruchmann, 2013; Wilson, Houston, Etling, & Brekke, 1996; Wu, Cheng, & Yen, 2012). In a perceptual anchoring paradigm, an analogy to knowledge (e.g., of the height of an object) may be the amount of visual information available, and the presence of more/better visual information may attenuate the anchoring effect by facilitating visual perception and judgment.

In the present study, we provide a particularly robust test of anchoring across two experiments using a VR‐based naturalistic perceptual task in which participants estimate the height of a virtual platform they are standing on. Critically, by engaging in an immersive task such as this, participants are directly sampling both lower‐level sensory and higher‐level cognitive information that informs their estimate in a naturalistic way. For example, moving the head gives lower‐level parallax cues to height, while higher‐level cues based on the heights of other features in the environment can also be used naturally. If anchoring persists, then it suggests there is at least potential for this phenomenon to impact on real‐world functional performance, which could have significant consequences (although it should be noted that biases in judgments such as this would not necessarily translate into consequences based on movement‐based interactions with the environment).

Our experiments have several important methodological features that combine many of the best aspects of previous research outlined above and lead to a particularly robust test of anchoring. First, consistent with Lee and Morewedge (2022), the task involves the participant estimating their height in the environment, based on information to which the participant has direct, online access (as opposed to estimating based on stored knowledge or relative to a recent memory of a stimulus). As noted above, if anchoring persists in such circumstances, then it is suggestive of a particularly robust phenomenon that cannot be overridden, even when the information that directly informs the estimate is immediately available. Second, in VR, we can vary parametrically the amount of information available in the environment. We assume that height estimates will improve as information quantity is increased, and so one might expect that increased estimate accuracy will reduce the anchoring bias. Such an effect would be analogous to previous work for higher‐level anchoring, suggesting that having more knowledge/familiarity with the topic leads to attenuation of anchoring effects (Smith et al., 2013). Third, in our VR task, the same participants can undertake many such height estimation trials with different anchors (both high and low). This contrasts with the vast majority of previous anchoring studies by enabling recovery of a within‐participant anchoring measure and at a range of anchor offset magnitudes. It also means that we can check for previously observed effects of the anchor offset (e.g., Mussweiler & Strack, 2001). Fourth, another benefit of the within‐participant anchoring measure is that we can also assess height estimates in the absence of an anchor. Doing so allows us to define both a subjective offset, as well as a subjective anchoring metric, both expressed relative to the participant's unanchored estimate of height as opposed to the objective veridical height, to which they do not have access. Finally, we also consider whether anchoring effects we observe are moderated by being in a 3D, naturalistic VR environment, comparing behavior in a similar non‐immersive condition.

To pre‐empt our results, our data suggest that anchoring does indeed persist in our immersive task and is not reduced relative to a non‐immersive equivalent. We also find that when the amount of information provided to make the estimate is increased and the accuracy of estimates improves accordingly, there is no accompanying reduction in anchoring effect; in fact, it increased. Finally, our alternative subjective anchoring measure is found to give rise to a particularly parsimonious description of the data.

## 2 Experiment 1

In Experiment 1, we examine perceptual anchoring during a height estimation task in VR. Estimating height involves the use of directly available (largely perceptual) information in the scene. This might involve estimates based on lower‐level perceptual cues to distance to the ground as well as higher‐level cues from the environment about height relative to other scene objects. Consequently, this reduces the contributions of pre‐existing knowledge (Epley & Gilovich, 2001) frequently involved in the classical cognitive anchoring paradigm as well as the impact of storing estimates in memory (Jain et al., 2021). In addition, we suggest that more visual information available in the environment will facilitate visual perception and estimation and therefore may attenuate anchoring. Also, the classical paradigm typically comprises a set of fixed questions with single answers and therefore requires a between‐subjects design since participants cannot experience the same question with both high and low anchors. In contrast, our approach enables us to ask the same participant to give a range of estimates involving matched trials with both high and low anchors and thus also affords calculation of an anchoring measure within each participant.

We hypothesize that estimation performance will improve when more perceptual information is present. We also hypothesize that since height estimation should get better with more perceptual information, the anchoring bias should be less evident in such circumstances.

### 2.1 Methods

#### 2.1.1 Participants

We recruited a total of 45 participants (*Female* = 25; *Male* = 20) from the University of Manchester. They were aged 18 or over (*M* = 25.29; *SD* = 5.93, range 18 to 39) years, able to stand unaided, and had normal or corrected to normal vision, wearing contact lenses, not glasses (since glasses interfere with VR headsets). The sample size was determined by a priori power analysis under the assumption of a medium effect size. Using “G*power” with 95% power and medium effect size (Cohen's *f* = 0.25) for a three‐way repeated‐measure ANOVA (Faul, Erdfelder, Buchner, & Lang, 2009), the associated sample size was around 45. This research received ethical approval from the University of Manchester Research Ethics Committee (Ref: 2022‐13479‐22431). Written informed consent was obtained from participants before experimentation.

#### 2.1.2 Apparatus

The experiment was conducted in VR using a 3D immersive environment built in house using the Unity3D Engine (Unity Technologies, 2018) and free assets available from SteamVR. We used an HTC VIVE head‐mounted display (HMD) to present the environment (via the SteamVR Unity plugin), and participants interacted with the experiment (and made responses) via a pair of HTC VIVE controllers. The HTC Vive has a resolution of 1080 × 1200 pixels per eye and field of view around 120 degrees. The HMD was connected wirelessly to a Stone PC using Windows 10 (i7 processor, 64‐Gigabyte RAM, Nvidia 1070 GTX GPU).

#### 2.1.3 Task

**Fig. 1:**
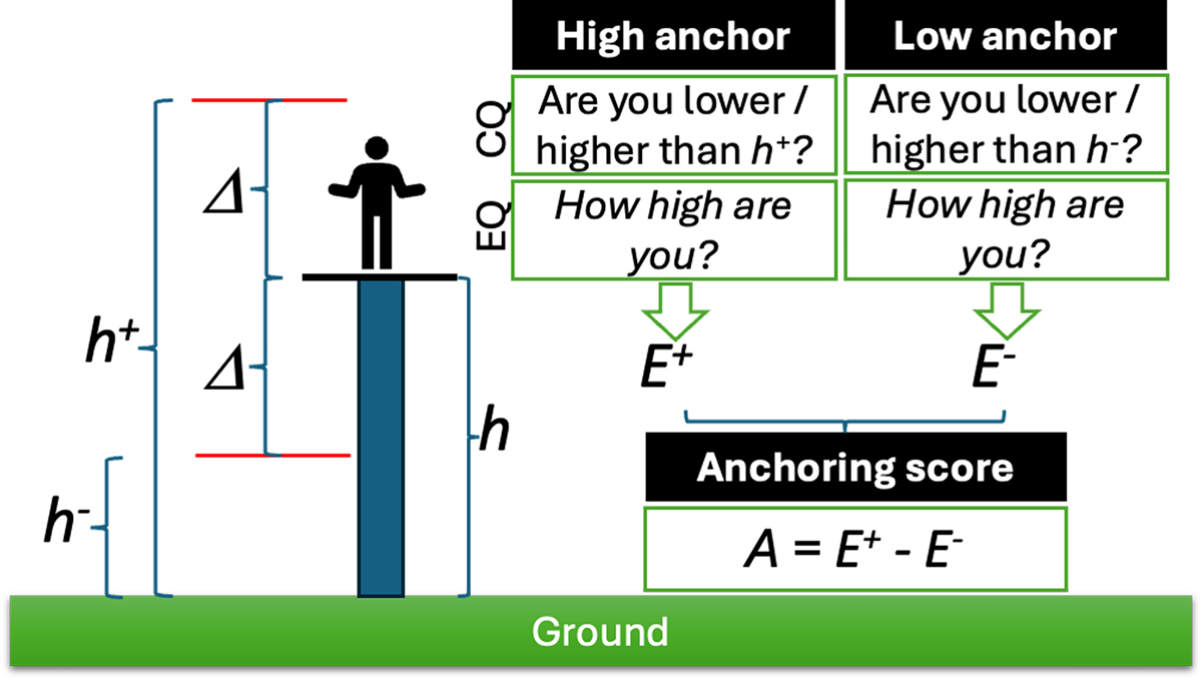
The schematic of the VR task and key variables. The participants stand on a virtual platform of height *h* m from the ground. They are first given a CQ and then an estimate question. For each height, there is a high‐anchor trial with the anchor of *h* ^+^ = (*h* + *Δ*) and a low‐anchor trial with the anchor of *h* ^−^ = (*h* ‐ *Δ*). The anchoring score (*A*) for a given height is calculated as the participant's estimate in the high‐anchor trial (*E* ^+^) minus the estimate in the low‐anchoring trial (*E* ^−^).

We employed an immersive perceptual form of the classical anchoring paradigm (see Fig. 1). Each trial consisted of one CQ and one EQ. The CQ required participants to make a relative judgment about whether the height of the platform on which they were standing was lower or higher than a provided “anchor” height (± *D* m from the platform height *h*). The estimate question followed and required participants to provide an estimate (in meters) of the height of the platform on which they were standing. If the CQ involved comparison with a higher anchor, the estimate was denoted *E_h_^+^*, whereas if it involved a comparison with a lower anchor height, it was denoted *E_h_^−^*.

#### 2.1.4 Design

**Fig. 2:**
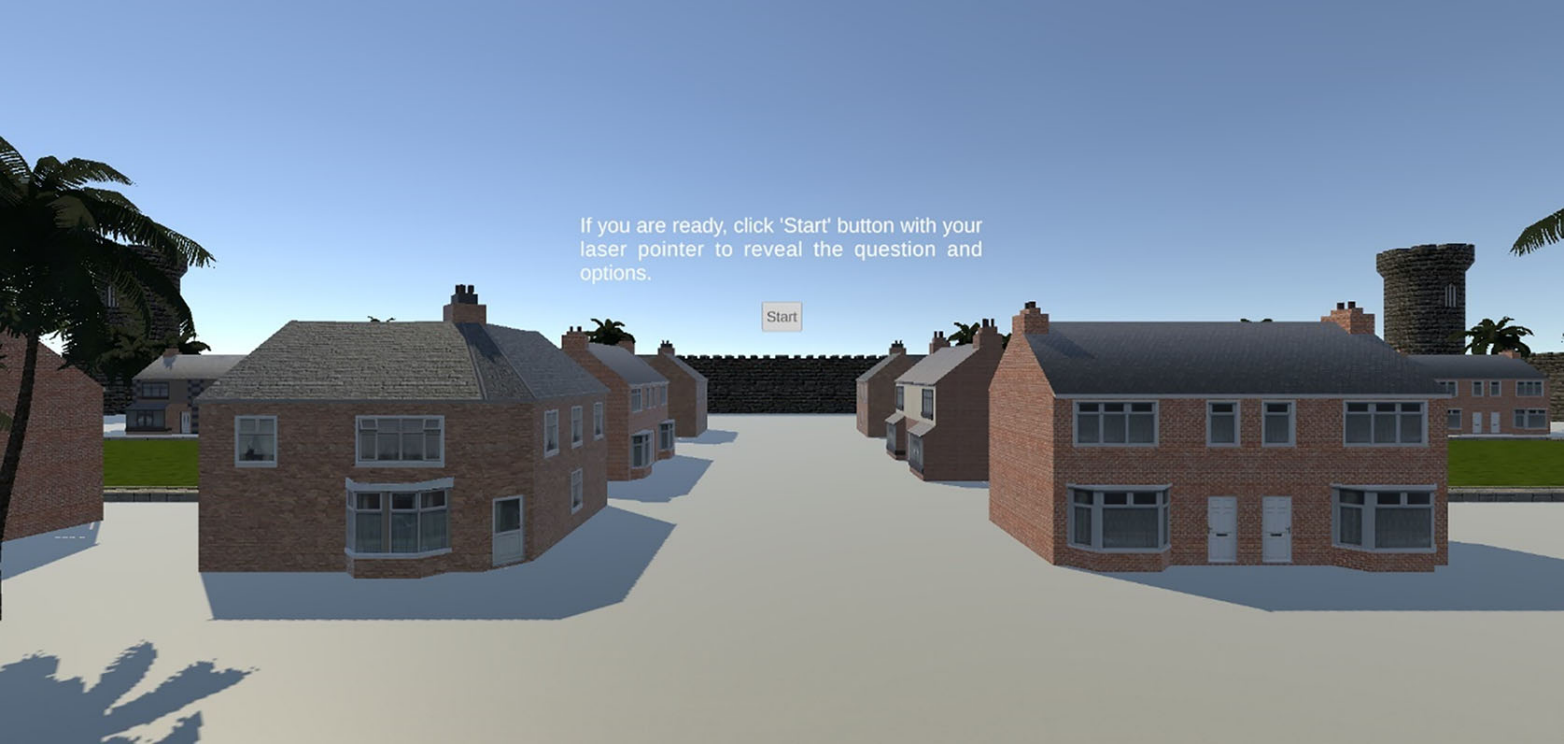
An example of the rich environment in the virtual reality (VR) task.

We manipulated three independent variables: (1) platform height (*h*): (6, 8, 10, 12, 14, 16 m), 2) anchor direction relative to the platform height (*D*): (−4 m, +4 m) and 3) environmental information (sparse/rich). The rich environment provided participants with rich visual information (to base their estimates) by including all designed objects as in Fig. 2, while the sparse environment included only city walls with lawns further away to minimize visual information (to a reasonable level that still provided some information on which to base a height estimate). Our dependent variables were:

1. The height estimates for each anchor direction (*E^−^*, *E^+^*);
2. from the height estimates obtained in different anchor direction conditions, it was subsequently possible to recover an anchoring measure, *A*, of the extent to which anchoring was observed (see Analyses section below and Fig. 1).

#### 2.1.5 Procedure

Participants were placed in VR with the experimenter's help. They acclimatized to VR for 3 min in the SteamVR home environment. They were then instructed to walk onto a real platform (a wooden board) raised approximately 2 cm above the ground, which was used to further enhance the sense of being on a real platform and thus immersion in the environment/task. Participants were asked to remain on this platform during the experiment. Once participants were ready, the experimenter launched the VR program. Participants were immediately placed in the experimental VR environment, standing on an elevated platform with houses, trees, and lawns underneath surrounded by city walls further away (see Fig. 2). In front of them, a question panel provided all the instructions and questions. Participants were instructed to press the “start” button using a ray emanating from the hand controller once they were ready to start and use the same process to interact with other buttons.

Participants then completed the comparative and estimate questions as described above. For the CQ, the participant saw the statement “Are you higher or lower than <anchor>m” accompanied by two possible answers (lower/higher) randomly allocated to the bottom left/right corners of the question panel. The estimate question was accompanied with a numeric pad for participants to input their estimate, and they used the same process of interacting with buttons to input and submit their estimates. When they finished a trial, they used the ray and trigger to click the “next” button to advance to the next trial. At the end of every trial, there was a 3 s “blind” period in which the scene was blocked from view in order to minimize the possibility of participants tracking the change of height from trial to trial.

Participants completed a total of 24 trials, corresponding to a factorial combination of the three independent variables: height (6), x anchor direction (2), x environment (2). Presentation order of trials was randomized with the additional constraints that: (1) trials of the same height were not adjacent; (2) rich and sparse environment trials were interleaved; (3) presentation order of the rich and sparse environments first was also counterbalanced over participants to minimize order effects.

#### 2.1.6 Analyses

We first summarized high‐level performance via the height estimates for the *i*th participant, (*E_i_^−^*, *E_i_^+^*), which were first averaged over participants and grouped by height, anchor direction, and environment. We also obtained an anchoring score for each participant by calculating the difference between estimates of the height in the high‐anchor trial versus that in the low‐anchor trial (see Fig. 1). Individual anchoring scores were then averaged over participants grouped by factorial combination of the levels of the platform height and environment variables.

Before conducting the inferential analyses, we first cleaned the data to minimize the impact of extreme values. Specifically, height estimates were first grouped by height, anchor offset, and environment. We then calculated the mean and median absolute deviation of estimates for each group. Finally, we excluded any trials for which the estimate was more than 2.5 median absolute deviations from the mean for that grouping. This process resulted in the exclusion of 67 estimates (6% of the total).

We examined the effect of platform height, anchor direction and environment on the height estimate using a three‐factor repeated‐measure ANOVA and subsequent Bonferroni‐corrected planned post hoc analysis. Evidence at the group level for an anchoring effect would be supported by significant effects of the anchor variable such that height estimates were higher in the high anchor condition.

### 2.2 Results

**Fig. 3:**
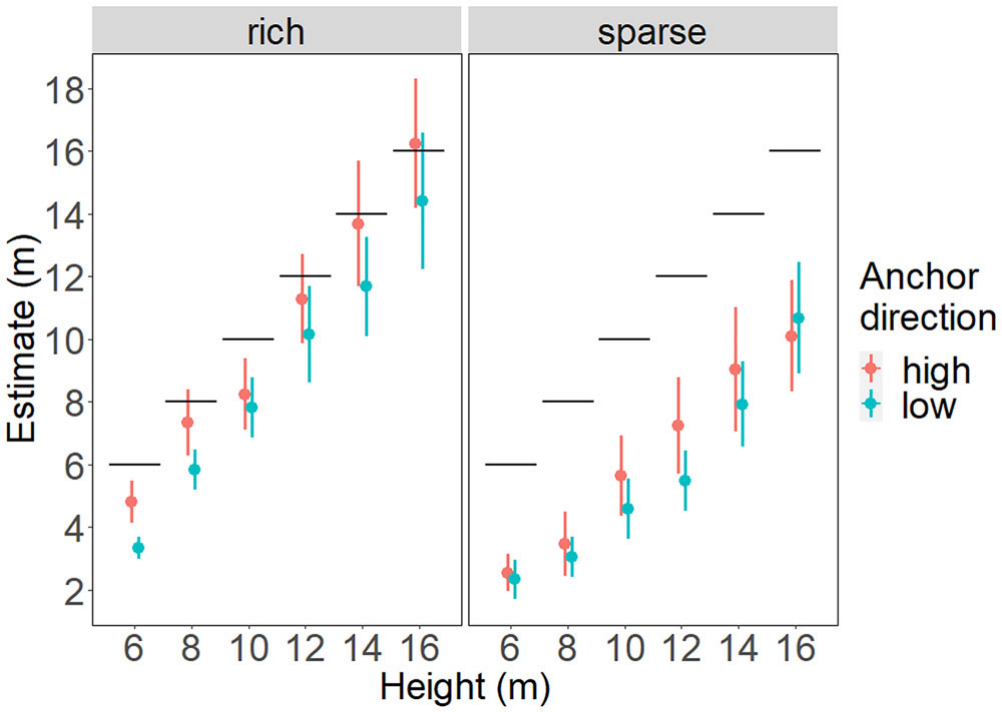
Means and confidence intervals of estimates for each height grouped by anchor direction and environment. Black dashes denote actual height. Error bars denote 95% confidence intervals.

After the exclusions outlined above, we conducted analysis on the remaining 1013 trials (94% of all trials). These data are illustrated in Fig. 3. Estimates increased monotonically with actual platform height and appeared to be more accurate in the rich environment relative to the sparse environment, suggesting that the provision of more information did indeed facilitate height estimation. Also, estimates appeared to be higher in high‐anchor trials relative to low‐anchor trials in both rich and sparse environments, suggesting the presence of the anchoring effect at the group level.

A three‐factor repeated‐measures ANOVA (height * anchor direction * environment) on height estimates showed significant main effects of height, *F*(1.80, 55.88) = 100.29, *p* < .001, *η_p_* ^2^ = 0.76, 95% CI = [0.67, 1.00], anchor direction, *F*(1,31) = 18.66, *p* < .001, *η_p_* ^2^ = 0.38, 95% CI = [0.16, 1.00], and environment, *F*(1, 31) = 60.25, *p* < .001, *η_p_* ^2^ = 0.66, 95% CI = [0.49, 1.00]. Also, it revealed significant interactions between anchor direction and environment, *F*(1, 31) = 9.46, *p* =  .004, *η_p_* ^2^ = 0.23, 95% CI = [0.05, 1.00], height and environment, *F*(3.30, 202.33) = 5.18, *p* =  .002, *η_p_* ^2^ = 0.14, 95% CI = [0.04, 1.00], and height, anchor direction, and environment, *F*(3.15, 97.60) = 4.16, *p* =  .007, *η_p_* ^2^ = 0.12, 95% CI = [0.02, 1.00].

To examine the simple effect of anchor within each environment, we compared responses for low versus high anchor conditions. In the sparse environment, the effect of anchor was not significant, *t*(31) = –1.04, *p* =  .31, with a mean difference of –0.25 (*SE* = 0.24). In the rich environment, however, there was a significant effect of anchor, *t*(31) = –5.40, *p* < .001, with a mean difference of –1.21 (*SE* = 0.22). Post hoc analysis showed that estimates were significantly higher in high‐anchor trials relative to low‐anchor trials for heights 6, 8, and 16 m (see Table 1) in the rich environment but for no heights in the sparse environment. Estimates were found to be significantly higher in the rich relative to the sparse environment in all 12 full factorial combinations of anchor direction and height (see Table 2).

**Table 1:** *t*‐values and associated significance levels for pairwise comparisons of height estimates between high‐ and low‐anchor trials split by environment and height

Environment	Height					
	6	8	10	12	14	16
Rich	3.66^***^	3.21^**^	0.51	1.84	1.76	4.01^***^
Sparse	0.58	0.42	0.85	1.61	1.15	−1.37

*Note*. ^***^ *p* < .001; ^**^ *p* < .01; ^*^ *p* < .05.

**Table 2:** *t*‐values and associated significance levels for pairwise comparisons of height estimates between rich and sparse environment trials split by anchor direction and height

Anchor Direction	Height					
	6	8	10	12	14	16
Low	2.97^**^	7.16^***^	7.61^***^	6.25^***^	4.64^***^	2.55^*^
High	4.83^***^	4.99^***^	5.56^***^	4.16^***^	4.54^***^	6.31^***^

*Note*. ^***^ *p* < .001; ^**^ *p* < .01; ^*^ *p* < .05.

**Fig. 4:**
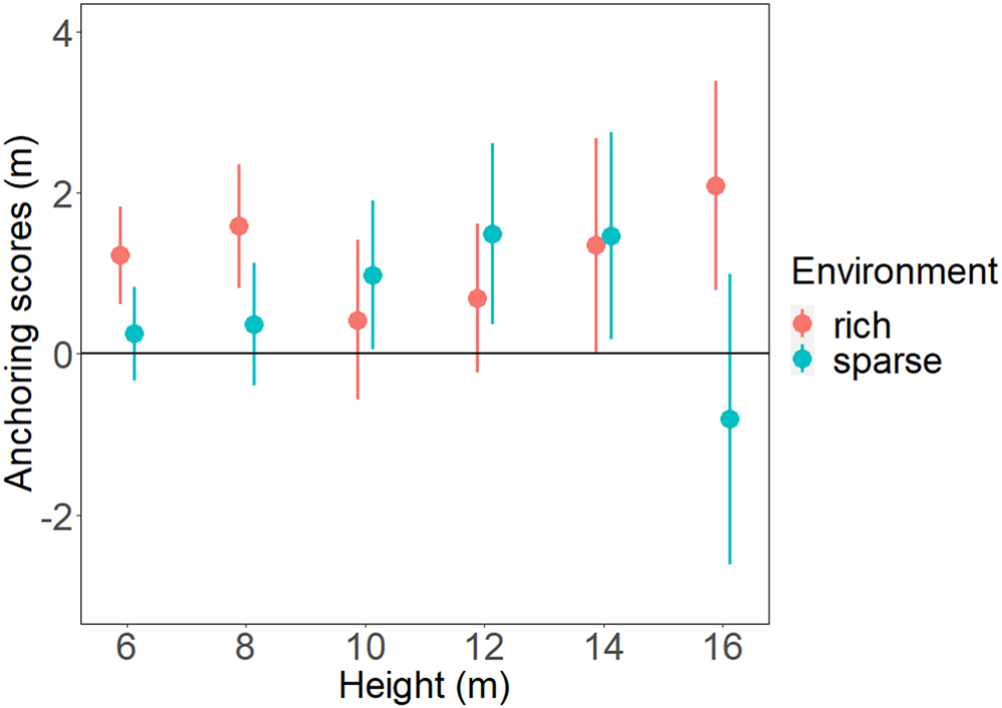
Means and confidence intervals of anchoring scores for each height grouped by environment. The black line at 0 refers to the absence of the anchoring effect. Error bars denote 95% confidence intervals.

Anchoring scores, calculated for paired trials within participants, were positive for every height and environment pair except one (Fig. 4). The exception was observed at height 16 m in the sparse environment. This is likely due to the particularly noisy responses seen in this condition, in which the participants were particularly high up, and there was sparse information available on which to base the estimate. Overall, the deviation of anchoring scores from 0 provided further evidence of the anchoring effect presenting at the individual level.

### 2.3 Discussion

The data from Experiment 1 suggest that a perceptual anchoring effect can be observed in a naturalistic height estimation task, even when perceptual information supporting the estimate is directly available, and participants are engaged in a 3D, immersive task. In addition, our design enabled us to measure anchoring within individuals. Moreover, we found compelling evidence that participants were basing their estimates on the available perceptual information (Fig. 3). However, the amount of anchoring did not appear to depend systematically on the amount of available information on which to base the estimate (Fig. 4). Anchoring effects seemed to be present for both sparse and rich scenes, but, in contrast to our prediction, these effects were stronger for the rich scene. These results suggest that the anchoring bias is a particularly robust and potentially generic phenomenon underpinning all estimation tasks and which persists in naturalistic activity.

Previous research has shown that the anchoring effect magnitude depends on the size of the anchor offset (Mussweiler & Strack, 2001; Teovanović, 2019; Wegener et al., 2001). If we are measuring aspects of the same mechanism in our VR‐based height estimation task, then we should anticipate similar effects. In our second experiment, we therefore manipulate the anchor offset magnitude. In addition, we also address the question of what we mean by an anchor offset (i.e., the difference between the true estimate and the anchor) in such experiments. Clearly, the participant does not know the true value they are trying to estimate, so this offset is not a measure that participants have access to. It may therefore be revealing to consider also how anchoring effects rely upon a subjective offset defined as the difference between the anchor and a subjective estimate. Consequently, we altered our design to enable recovery of an alternative measure of the anchoring effect, which is calculated relative to a subjective estimate as opposed to the objective “correct” answer.

It also remains unclear whether our anchoring effects have at all been attenuated by being engaged in a 3D, immersive, naturalistic VR task. To investigate this question, we also examine behavior in a more traditional task in which stimuli were delivered via a 2D display.

## 3 Experiment 2

Experiment 1 suggests that anchoring persists when we have direct access to perceptual information in an immersive VR task. In Experiment 2, we consider whether these effects depend on the distance from the true height to the anchor height (the offset) as has been shown in previous anchoring studies (e.g., Mussweiler & Strack, 2001). Critically, we also included trials where participants were asked to estimate their height without an anchor (i.e., participants only saw and responded to the EQ with no preceding CQ). This subsequently allowed us to recast our data in terms of a *subjective anchor offset* (SAO) and the *subjective anchoring score* (SAS), which take into account individual differences in perceived height. Finally, to assess the extent to which anchoring effects are affected by the VR presentation, we also introduced a 2D non‐immersive version of the height estimation task.

### 3.1 Methods

#### 3.1.1 Participants

We recruited a total of 127 participants (*Female* = 105; *Male* = 22; *VR* = 63; *2D* = 64) from the University of Manchester. All participants were aged 18 or over (*M* = 19.12; *SD* = 0.94, range 18 to 22) years, could stand unaided, and had normal or corrected to normal vision wearing contact lenses but not glasses since glasses can be uncomfortable to wear in VR headsets or damage the optics. The sample size for each condition (2D/VR) was based on that of Experiment 1 (*N* = 45). We aimed to recruit at least this many participants in each comparable case. In practice, this meant ensuring a sample size of at least 45 for both levels of our between‐participants factor (see Design section below), which was achieved with our sample size of *N* = 127.

#### 3.1.2 Apparatus

In the VR condition, the HMD and the PC equipment were the same as in Experiment 1. In the new 2D conditions, static images from the VR headset were presented on a 24″ LCD display (Iiyama ple2483h) with resolution 1920 × 1080 at 60 Hz. To ensure we had approximately equal numbers in each group throughout the experiment, participants were alternately placed in either the 2D or VR experiment in order of recruitment.

#### 3.1.3 Design

We employed a mixed design with three within‐subjects independent variables: platform height (10, 12, 14 m), anchor direction (low, high), and anchor offset (0, 2, 4, 8 m). Note that 0 anchor offset meant that there was no anchor at all, and participants simply estimated the platform height without a CQ. The fourth, between‐subjects, independent variable was display (2D/VR). In all conditions, we used the rich environment from Experiment 1. We measured a number of dependent variables including three also used in Experiment 1, namely, height estimate, response accuracy in the CQ, and anchoring score. We also introduced two further variables: SAS and SAO. For details of how these variables were calculated, see the Analyses section below.

#### 3.1.4 Procedure

The procedure for the VR condition remained the same as Experiment 1, and this was also repeated for the 2D version. Participants allocated to the 2D version ran the 2D counterpart of the same program on a desktop PC and used a mouse to respond. They were seated approximately 50 cm from the display, but the head was not restrained, and they were allowed to move freely. In total, participants in both VR and 2D tasks completed 24 trials corresponding to the full factorial combination of the three within‐participant independent variables outlined above.

#### 3.1.5 Analyses

In addition to the analyses outlined in Experiment 1. We calculated two further metrics, namely, SAO and SAS:

- SAO was calculated for participant *i* on each trial as the anchor height minus the estimate in the corresponding non‐anchored trial, so, for example, on a high anchor trial at height *h^+^*, the SAO is Δ *_i_* (*h*) = *h^+^ − E_i_^0^(h)*.
- SAS was calculated as the difference between the estimate of the height in an anchored trial and the estimate of height in the corresponding non‐anchor trial. Therefore, for a high anchor trial with height estimate *E_i_^+^* (*h*), the SAS is *A_i_(h) = E_i_^+^(h) − E_i_^0^* (*h*). Note that this measure provides an anchoring estimate from a single trial (relative to the unanchored estimate).

Similar to Experiment 1, we examined the effect of platform height, anchor direction, anchor offset, and display on height estimates using a four‐factor repeated‐measures ANOVA with subsequent Bonferroni‐corrected post hoc analysis. Again, evidence of anchoring on the group level would be supported by significant effects of the anchor variable such that height estimates were higher in the high anchor condition.

We then examined anchoring on the individual level (using the measure outlined in Experiment 1) across each of the platform height × display conditions.

Turning to our subjective measures, we hypothesized that as subjective offsets increased, the subjective anchoring measure should also increase. Consequently, we conducted mixed‐effect regressions to examine the extent to which SAOs predicted SAS. We included two fixed effects (SAO and display condition) and one random effect (participant).

### 3.2 Results

**Fig. 5:**
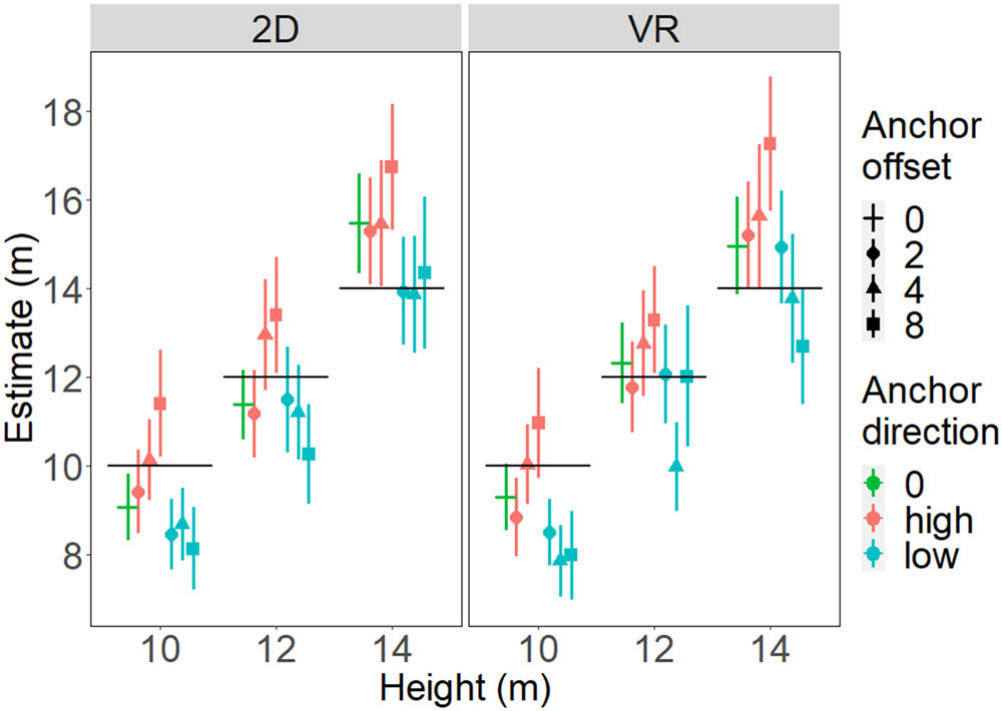
Means and confidence intervals of estimates for each height grouped by anchor direction, anchor offset, and display. Black dashes mark the real actual height. Error bars denote 95% confidence intervals.

We excluded 224 trials (7% of the total) from the data according to the criteria outlined in the methods of Experiment 1 and conducted analysis on the remaining 2824 trials. The summary of estimates showed a similar pattern between 2D and VR conditions (Fig. 5). Similar to Experiment 1, estimates increased monotonically with actual heights. Also, estimates were comparable with actual heights in both the 2D and VR displays. Consistent with Experiment 1, estimates were higher in high‐anchor trials relative to low‐anchor trials, suggesting the presence of anchoring on the group level. In addition, for both low and high anchors, there was a tendency for estimates to be biased further from the actual height with increasing offset (although this tendency was clearer for high anchor data).

A four‐factor (three within, one between) ANOVA (height * anchor direction * anchor offset * display) on estimates was conducted. It revealed significant main effects of height (*F*(1.45, 122.21) = 271.49, *p* < .001, *η_p_* ^2^ = 0.76, 95% CI = [0.71, 1.00]), anchor direction (*F*(1, 84) = 96.85, *p* < .001, *η_p_* ^2^ = 0.54, 95% CI = [0.42, 1.00]), and a significant interaction between anchor direction and anchor offset (*F*(1.88, 158.05) = 20.36, *p* < .001, *η_p_* ^2^ = 0.20, 95% CI = [0.11, 1.00]). All other factors were non‐significant. Post hoc analysis on the main effect of height showed that estimates were the highest on height 14 m, followed by height 12 m and height 10 m (Table 3). Post hoc analysis on the interaction between anchor direction and anchor offset (Table 4) showed that estimates were higher in the high‐anchor trials relative to low‐anchor trials with all three anchor offsets. On the other hand, estimates were higher with the anchor offset of 2 m relative to 8 m in the low‐anchor trials (Table 5).

**Table 3:** The *t*‐values and associated significance levels of pairwise *t*‐tests on estimates between heights

Contrast		
10–12	10–14	12– 14
−13.60^***^	−18.60^***^	−14.20^***^

*Note*. ^***^ *p* < .001; ^**^ *p* < .01; ^*^ *p* < .05.

**Table 4:** The *t*‐values and associated significance levels of pairwise *t*‐tests on height estimates between high‐ versus low‐anchor trials split by anchor offset

Contrast	Anchor Offset		
	2	4	8
High anchor – low anchor	4.39^***^	7.23^***^	8.51^***^

*Note*. ^***^ *p* < .001; ^**^ *p* < .01; ^*^ *p* < .05.

**Table 5:** The *t*‐values and associated significance levels of pairwise *t*‐tests of height estimates between anchor offsets anchor direction

Anchor Direction	Contrast		
	2–4	2–8	4–8
High	−1.98	−5.17^***^	−2.82^*^
Low	1.41	3.30^**^	2.11

*Note*. ^***^ *p* < .001; ^**^ *p* < .01; ^*^ *p* < .05.

**Fig. 6:**
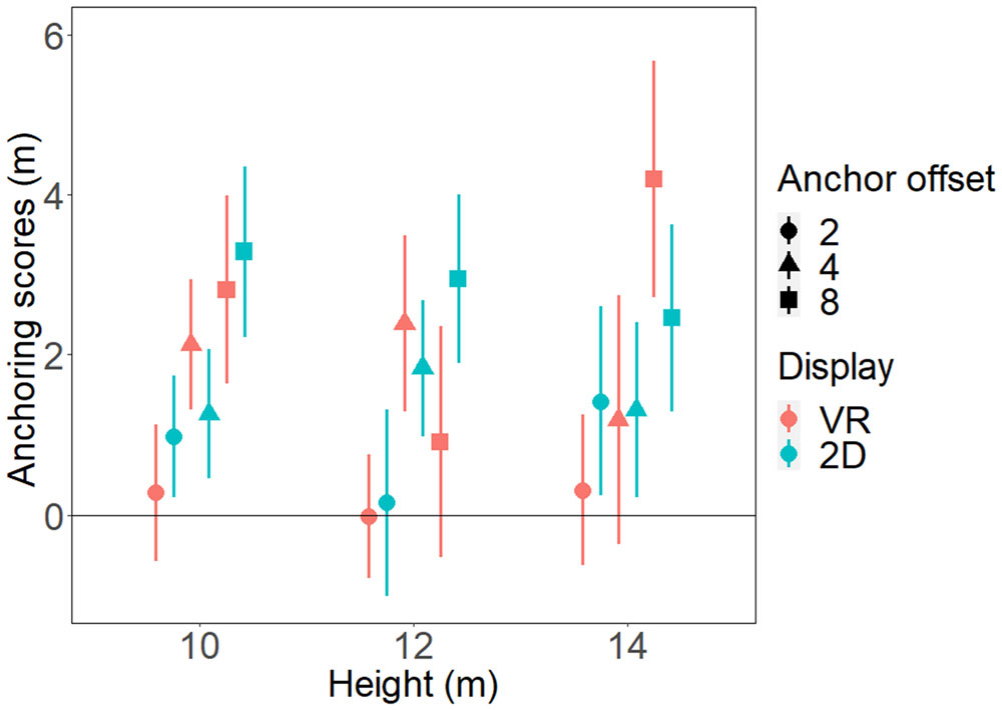
Means and confidence intervals of anchoring scores for each height, grouped by anchor offset and display. The black line at 0 refers to the absence of anchoring effect. Error bars denote 95% confidence intervals.

At the individual level, the mean anchoring scores summarized from paired trials within participants were all positive or nearly positive over 2D and VR conditions (Fig. 6). Overall, the results reinforced the presence of anchoring effect on the individual level and indicated the positive relationship between anchor offsets and anchoring effect.

**Fig. 7:**
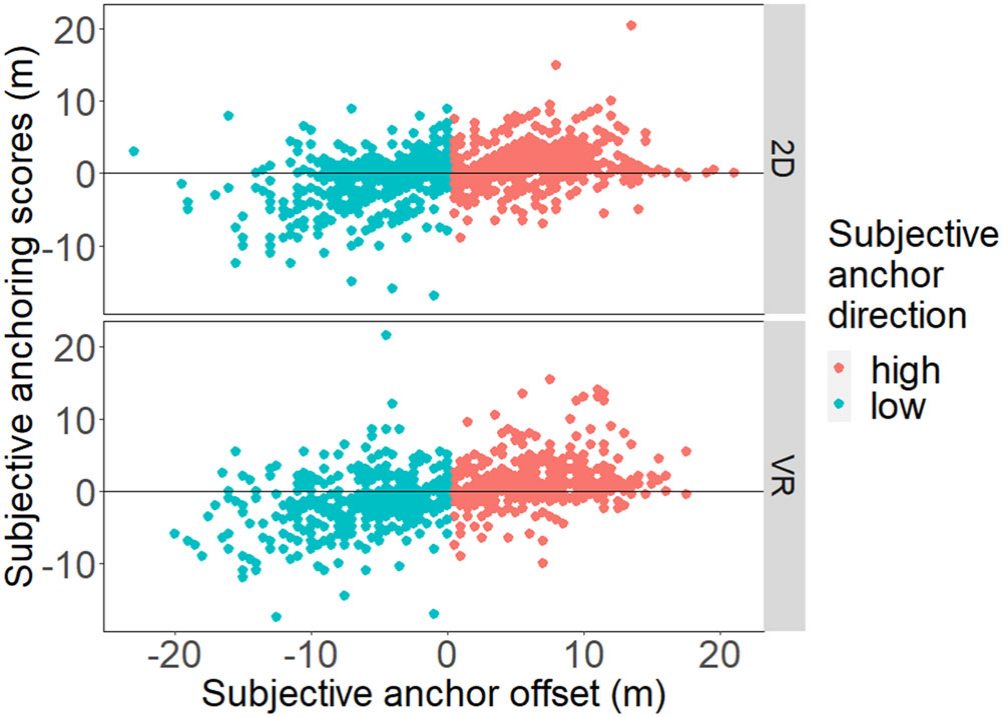
The point plot of subjective anchoring scores (SASs) against subjective anchor offsets (SAOs) colored by subjective anchor direction.

In Fig. 7, we present SASs across all trials as a function of the SAO. As anticipated, we observed a positive relationship between these variables. The pattern was similar for both VR and 2D conditions.

A linear mixed‐effect regression was conducted to test if SAO and presentation type predicted SASs. The results showed that SAO was a significant predictor, *β* = 0.66, *t*(1904.85) = 42.69, *p* < .001, *η_p_* ^2^ = 0.73, 95% CI = [0.72, 1.00], supporting the idea that SASs are predicted by SAOs. More specifically, when the subjective offset is large and negative (i.e., below the perceived platform height), the subjective anchoring effect is also large and negative (i.e., the estimate is biased toward the low anchor). This negative bias gradually reduces to close to 0 as the subjective offset approaches 0. As the subjective offset becomes more positive, the subjective anchoring effect continues to increase (i.e., the estimate is biased toward the high anchor). Also, the regression showed a significant interaction between SAO and display, *β* = 0.13, *t*(1878.27) = 6.22, *p* < .001, *η_p_* ^2^ = 0.02, 95% CI = [0.01, 1.00], suggesting that the rate of increase in SAS with subjective offset was slightly faster in the VR condition.

### 3.3 Discussion

We found very similar effects irrespective of whether the task was carried out in immersive 3D VR or using more traditional 2D display conditions. This suggests that anchoring persists even when the information used to underpin the estimate is picked up via naturalistic, dynamic interaction with the environment. Anchoring effects also displayed similar dependence on height and objective offset. In addition, we found evidence that anchoring depended approximately linearly on offset and, similarly, subjective anchoring depended linearly on the subjective offset (both in VR and 2D display conditions). Taken together, these data are consistent with Experiment 1 and previous research suggesting that the magnitude of anchoring is affected by offset magnitude. We conclude that the effects observed in our two experiments are likely to be based on similar underlying mechanisms to those implicated in previous anchoring studies. We also suggest that these data indicate a generic mechanism that underpins all anchoring phenomena. In the next section, we show that the subjective offset and anchoring measures described above allow a particularly parsimonious re‐description of anchoring as a linear combination of an unanchored estimate and the anchor value itself.

## 4 Data modeling

Here, we provide a simple model of our data, starting from the premise that participants make biased and noisy estimates of the actual height h on all trials (either anchored or unanchored). We emphasize at the outset that this might be considered a simple redescription of the data; however, in doing so, we provide a parsimonious account that is only possible because we have an estimate of perceived height when no anchor is present. This approach is also broadly in line with the anchor integration model (AIM) proposed by Turner and Schley (2016). In that paper, it is proposed that anchoring type effects might arise from Bayesian combination of a prior distribution characterizing the participant's underlying (unanchored) noisy estimate together with another noise‐corrupted distribution based on the anchor. Ultimately, this gives rise to an estimate that is a weighted linear combination of prior and anchor estimates, with the relative contributions of the anchor and the prior to the final estimate determined by the relative amounts of noise in the two information sources. Our approach is similar in that it assumes a weighted linear combination of anchored and unanchored estimates. However, we cannot explicitly measure the noise on the anchor information here (which would determine its weight in the linear combination under Bayesian updating), so we fit our data using a different (admittedly simpler) approach that suits the design of our experiment. Note also that our experimental design and model formulation enable us to model subjective anchoring effects as described above.

### 4.1 The subjective anchoring model (SAM)

We assume that participant i’s mean estimate of height h is ${\underline{E}}_{i^{0}\left(h\right)}$, which can be modeled as a sample from a biased Gaussian population:

(1) $${\underline{E}}_{i}^{0}\left(h\right)∼N\left(h+\beta_{pop}\left(h\right),\sigma_{pop}^{2}\left(h\right)\right),$$

or equivalently

$${\underline{E}}_{i}^{0}\left(h\right)=h+\beta_{pop}\left(h\right)+\varepsilon_{pop}^{i}\left(h\right)$$

where $\varepsilon_{pop}^{i}\left(h\right)∼N\left(0,\sigma_{pop}^{2}\left(h\right)\right)$.

We then assume that on trial j, participant i makes a height estimate $E_{ij}^{0}\left(h\right)$, which is centred on the mean value described in (1):

(2) $$E_{ij}^{0}\left(h\right)∼N\left({\underline{E}}_{i^{0}}\left(h\right),\sigma_{est}^{2}\left(h\right)\right),$$

or equivalently

$$E_{ij}^{0}\left(h\right)={\underline{E}}_{i^{0}}\left(h\right)+\varepsilon_{est}^{ij}\left(h\right)$$

where $\varepsilon_{est}^{ij}\left(h\right)$ $∼N(0,\sigma_{est}^{2}\left(h\right))$.

We suggest that the participant makes a height estimate as specified by (2) on all trials. However, on anchored trials, we suggest that such an estimate is linearly combined (via a weighting factor λ) with the anchor height $h^{a}$, to give the anchored estimate $E_{ij}^{a}\left(h\right)$:

(3) $$E_{ij}^{a}\left(h\right)=\lambda h^{a}+\left(1-\lambda \right)E_{ij}^{0}\left(h\right)(0<\lambda <1$$

This constrains the anchored estimate to be between the unanchored estimate and the anchor height on trial j. When parameter λ is 0, the anchored estimate is equal to the unanchored estimate, and when λ is 1, the anchored estimate is equal to the anchor. For all other values, the anchored estimate is between these extremes. Note also that the expression for the anchored estimate in (3) makes it clear that the variance of anchored estimates is given by:

(4) $$var\left(E_{ij}^{a}\left(h\right)\right)=var\left(\left(1-\lambda \right)E_{ij}^{0}\left(h\right)\right)={\left(1-\lambda \right)}^{2}var\left(E_{ij}^{0}\left(h\right)\right).$$

A clear corollary of this is that the variance for the anchored trials must be smaller than that for the unanchored trials and also that the weighting factor can be estimated from the variances in anchored and unanchored trials:

(5) $$\lambda =1-\sqrt{\frac{var\left(E_{ij}^{a}\left(h\right)\right)}{var\left(E_{ij}^{0}\left(h\right)\right)}}.$$

Rearranging (3), we see that it can also be rewritten as

(6) $$A_{ij}\left(h\right)=\lambda \Delta_{ij}\left(h\right)$$

where

$$\begin{array}{ccc} A_{ij}\left(h\right) & = & E_{ij}^{a}\left(h\right)-E_{ij}^{0}\left(h\right)\\ \Delta_{ij}\left(h\right) & = & h^{a}-E_{ij}^{0}\left(h\right) \end{array}$$

are, respectively, the SAS and the SAO as defined in Section 3.1. From (6), it is clear that, on trial j, the subjective anchoring effect is then a simple scalar multiple of the subjective offset.

Turning to our experiment, note that we do not have access to $E_{ij}^{0}\left(h\right)$ on anchored trials since we only measure $E_{ij}^{a}\left(h\right)$. However, due to our design, we can approximate $E_{ij}^{0}\left(h\right)$ simply as the estimate recovered in our unanchored trials. More explicitly, for all trials with participant i, we have replaced $E_{ij}^{0}\left(h\right)$ in (4) with the height estimate $E_{i^{0}}\left(h\right)$ recovered by that participant in the unanchored trials (or the mean of the two estimates provided to be more precise). This is precisely how we have defined the SAS and SAO in Section 3.1 (and plotted in Fig. 7) where

(7) $$\begin{array}{ccc} A_{ij}\left(h\right) & = & E_{ij}^{a}\left(h\right)-E_{i}^{0}\left(h\right)\\ \Delta_{i}\left(h\right) & = & h^{a}-E_{i}^{0}\left(h\right) \end{array}$$

are, respectively, the experimental SAS and the experimental SAO.

### 4.2 Model fitting and data simulation

We fit the model with a single free parameter (i.e., the weighting parameter λ) to the mean participant height estimates in each of the 18 anchored trials from the VR display condition in Experiment 2. To do this, we first set each of $\sigma_{pop}^{2}\left(h\right)$ and $\sigma_{est}^{2}\left(h\right)$ to zero so that the minimization process was not subject to stochastic variations, which would have led to local minima and an unstable fit. We measured $\beta_{pop}\left(h\right)$ directly from our data, and values for this parameter are provided in Table 6. The model was developed in MATLAB (MathWorks, Inc., 2022) and fitted to the data using the MATLAB function fminsearch, which implements the Nelder‐Mead simplex algorithm, as described in Lagarias, Reeds, Wright, and Wright (1998).

**Table 6:** Parameter estimates measured from our dataset

Parameter	h = 10	h = 12	h = 14
$\beta_{pop}\left(h\right)$	−0.61	−0.02	1.24
$\sigma_{pop}^{2}\left(h\right)$	16.49	24.51	33.58
$\sigma_{est}^{2}\left(h\right)$	3.59	6.35	11.31

The best fitting value of λ recovered from our fitting process was 0.1931. This figure suggests that the mean height estimates observed are consistent with a weighted linear combination of the given anchor (around 20% weighting) and an unanchored height estimate (around 80% weighing). The anchored estimate is consequently significantly closer to the unanchored estimate than the anchor.

**Fig. 8:**
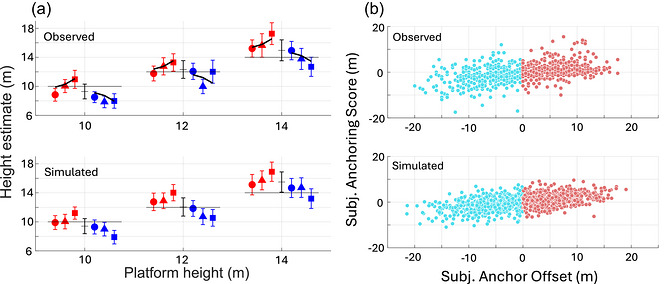
a) Height estimate data from Experiment 2 VR display condition (top panel) together with the best fitting (λ = 0.1931) noise‐free model (black lines). Bottom panel shows the simulated data using the same value of λ and the noise parameters in Table 6. Error bars denote 95% CIs. (b) SAS versus SAO for observed and simulated data (note top panel is the same data as presented in Fig. 7).

**Fig. 9:**
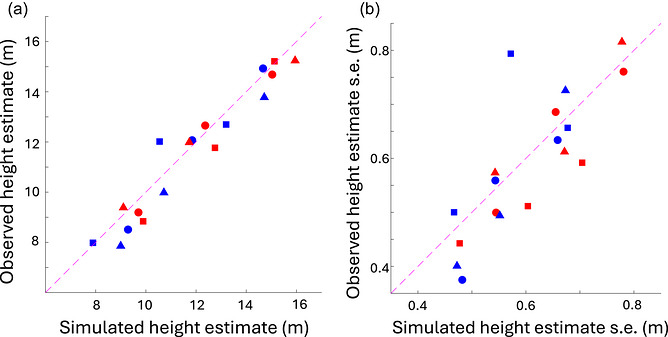
Simulated versus observed mean (a) and *s.e*. (b) of height estimates recovered for the simulated data.

The outcome of this fit (with no noise) is shown as the solid black lines in Fig. 8a (top panel). Using the fitted λ value, we can also generate data for a sample of 62 simulated participants by incorporating measured variability parameters $\sigma_{pop}^{2}\left(h\right)$ and $\sigma_{est}^{2}\left(h\right)$ as calculated from our data. For $\sigma_{pop}^{2}\left(h\right)$, we calculated the average variance over participants in the unanchored trials at each height. For $\sigma_{est}^{2}\left(h\right),$ we calculated the within‐observer variance over repeated unanchored trials and then averaged this over participants at each height. The recovered values are given in Table 6. Using these parameters in the model together with the values for $\beta_{pop}\left(h\right)$ and the fitted weighting parameter, we simulated the data shown in Fig. 8a (bottom panel). Fig. 8b illustrates that the relationship between SAO and SAS is similar for both observed (top panel) and simulated (bottom panel) data. Note that both the mean and variability in the simulations presented in Fig. 8a are well matched to those of the observed data. To see this more clearly, we have plotted fitted versus observed mean and *s.e*.s in Fig. 9.

**Fig. 10:**
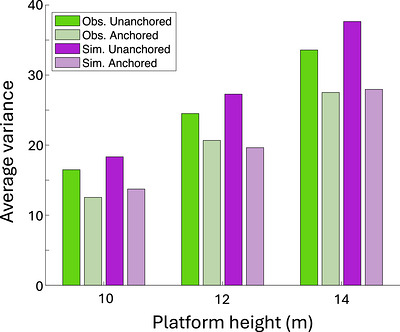
Variance comparison for observed and simulated data for anchored and unanchored trials across the heights. Anchored values are averaged across the offset conditions.

In (4) above, we show that under the SAM model, the variance in an unanchored condition should be higher than in the corresponding anchored condition. Fig. 10 shows that this is the case for both the simulated and the observed data. We used (5) to make a direct estimate of λ from the variance data at each height. The average estimate for λ across heights was 0.1014. While this is lower than the value obtained from the fitting procedure outlined above, it is consistent with the idea that the anchor is given considerably less weight than the estimate in the linear combination.

## 5 General discussion

In Experiment 1, we provide evidence that anchoring persists in a naturalistic 3D immersive VR‐based task in which participants estimate height^1^ using relevant, direct information present in the environment. Due to our novel paradigm, we were able to present evidence for anchoring via both between and within participant analyses. Critically, even though we found evidence that the information was being used to inform height estimates (because more information led to better estimates), the strength of the anchoring effect did not appear to depend on the amount of information available.

In Experiment 2, we systematically modified the anchor offset direction and magnitude and measured anchoring effects (and offsets) relative to a subjective estimate of platform height. We replicated our findings from Experiment 1 and also present evidence that anchoring scores in this task depend upon the anchor offset direction and magnitude, broadly increasing as the anchor offset increases, in line with previous research (Mussweiler & Strack, 2001; Teovanović, 2019; Wegener et al., 2001). Finally, based on our subjective measures, we present a particularly parsimonious account of our data in which the height estimate is captured as a linear combination of the anchor and an unanchored estimate.

### 5.1 The robustness and universality of anchoring effects

As noted in the Introduction, many previous studies have noted that anchoring is a robust bias phenomenon that can be observed in both lower and higher level cognitive processing (e.g., Jacowitz & Kahneman, 1995; Jain et al., 2021; LeBoeuf & Shafir, 2006; Spicer et al., 2022). Our data are consistent with this and further support data from previous studies (e.g., Lee & Morewedge, 2022), suggesting that anchoring persists even when participants have direct, online access to the information required to make the estimate. Our study, however, also provides an even more robust test of anchoring, suggesting it also persists when the manner in which participants sample information in order to make the estimate is naturalistic, dynamic, and close to that in the real world. Taken together with previous research, this suggests that anchoring is a highly generic mechanism, which is a universal feature of any estimation task, irrespective of the characteristics of that task.

### 5.2 The dependence of anchoring on offset magnitude

Several previous studies (Mussweiler & Strack, 2001; Teovanović, 2019; Wegener et al., 2001) have found evidence for changes in anchoring scores as a function of anchor offset magnitude. Our data are consistent with these studies but extend the results to a quasi‐realistic task, involving naturalistic information sampling. There is also data showing that with more extreme anchor offsets, anchoring effects persist, though they are greatly reduced relative to more moderate offsets (Mussweiler & Strack, 2001; Strack & Mussweiler, 1997; Wegener et al., 2001; Wegener, Petty, Blankenship, & Detweiler‐Bedell, 2010). This reduction suggests that there are limits beyond which the brain is no longer willing to take the anchor into account, and it is disregarded. We did not observe such a reduction at the largest offsets tested in our data, potentially because our offsets were not sufficiently extreme (note also that our offset choices were bounded in magnitude due to having a hard limit below from the floor).

### 5.3 Subjective anchoring metrics and the SAM account

The subjective metrics (SAO and SAS) presented offer a novel way to characterize anchoring measurement in paradigms where it is possible to get a baseline subjective measure of the estimate in question (clearly, this is not possible for most classical anchoring paradigms). Crucially, this enabled us to recover an anchoring measure for each participant in each trial. Moreover, after recoding the data in this way, we arrived at a particularly parsimonious and summary of the data in which SAO and SAS are linearly related. This linear relationship between SAO and SAS is also consistent with the SAM account presented in Section 4, which describes anchoring effects as a weighted linear combination (on each trial) of the given anchor height and an unanchored subjective estimate of height. We do not have any strong evidence for SAM as a generative model of anchoring, but, as noted above, it is consistent with the Bayesian AIM of Turner and Schley (2016). Bayesian accounts are clearly prevalent in the fields of cognition and perception generally. With respect to perception, humans have been shown to (approximately) linearly combine sensory information, both within and across the senses, in situations where multiple, redundant sources are available. For example, there is evidence for an optimal weighted linear combination of noisy sensory signals for estimation of size from vision and touch (e.g., Ernst & Banks, 2002), position from vision and hearing (Allais & Burr, 2004) and motion from retinal and extra‐retinal input (Freeman, Champion, & Warren, 2010; Gu, Angelaki, & DeAngelis, 2008). Similarly, optimal linear combination of noisy information sources has been suggested as a potential account of higher‐level cognitive phenomena such as contextual preference reversals (Howes et al., 2016) and, indeed, anchoring (Lieder, Griffiths, Huys, & Goodman, 2018; Turner & Schley, 2016). In Bayesian update models, a combination of a prior with new information occurs in a manner similar to Eq. (3), with the additional constraint that the weights are determined by the precision of the information sources, with more precisely encoded information given greater weight in the combination (e.g., see Ernst & Banks, 2002, for details). We make no claims about such a constraint in the SAM model. Directly measuring the precision (subjective or objective) of the information provided by the different information sources was not possible in our experiment. Nonetheless, finding evidence that anchoring is based on an optimal combination of available information would suggest that such accounts might represent a generative model of anchoring behavior, explained by an optimal combination of multiple, noisy information sources in order to maximize the precision of the integrated estimate.

### 5.4 The potential for anchoring to impact on real‐world behavior

In Experiment 2, we found limited evidence for differences in VR versus a more traditional screen‐based presentation of the experiment. Importantly, these data and those from Experiment 1 suggest that anchoring does not go away when judgments are made in naturalistic tasks. The kinds of experiments we conduct in laboratory settings often involve highly abstracted scenarios and do not necessarily require the participant to pick up task‐relevant information in a natural way. In our task, however, the height estimates are recovered from actively engaging with the available information in the scene, for example, looking around to gather information from the surroundings about cues to height such as object size and stereo disparity. That the effects persist in these circumstances emphasizes that the anchoring bias could be genuinely impactful in real‐world tasks and highlights the need to identify potential scenarios where such impacts could be problematic. It is worth noting here that the biases observed were in reported quantitative estimates. If one were in a task in which performance depended on such an estimate, then it is possible that such biases would lead to problems. Biases in perception are not necessarily shared by the systems that govern action (e.g., see Bingham & Pagano, 1998; Pagano & Bingham, 1998). So, tasks in which performance depends on motor activity may then not be affected unless such biases are shared between perceptual‐motor systems. With this in mind, there is clearly scope for future experiments to address this issue.

## 6 Conclusion

The present study presents compelling evidence that anchoring persists in a 3D immersive VR task in which estimates are based on perceptual and cognitive information that is directly available to the observer and sampled in a naturalistic way, consistent with real‐life estimation tasks. We speculate that anchoring is a generic feature of any estimation task and underlying (biased) processing is common across all such tasks. We also provide a new within‐subjects anchoring paradigm that enables a subjective assessment of anchoring, taking account of individual differences in estimation. Using this metric, we provide a parsimonious account of anchoring based on simple linear combinations of available information. Taken together, these results emphasize the potential for this cognitive bias to have a significant impact on performance in any real‐world task requiring quantitative estimation.

## Conflict of Interest Statement

The authors declare no conflict of interest.

## Data Availability

All research materials, data, and analysis script are made publicly available at the Open Science Framework and can be accessed at [https://osf.io/jn4hw/?view_only=dd0ac66794fd47abaa03329e32408507]. Data analysis was carried out using R 3.6.3 (R Core Team, 2020).

## Note

1 Note that participants were instructed to report platform height. It is possible, however, that they ignored these instructions and instead reported eye height. We do not believe they did this, but even if they did, it would have simply added a bias, together with additional noise in line with the variability in adult height (*s.d*. ≈ 0.075 m). Given that estimates in our rich conditions in Experiment 2 were largely accurate and 1–2 orders of magnitude larger than the *s.d*. in adult height, we feel it is highly unlikely that this would impact on our results.
